# Prognostic value of aerobic capacity and exercise oxygen pulse in postaortic dissection patients

**DOI:** 10.1002/clc.23537

**Published:** 2020-12-31

**Authors:** Pascal Delsart, Camille Delahaye, Patrick Devos, Olivia Domanski, Richard Azzaoui, Jonathan Sobocinski, Francis Juthier, Andre Vincentelli, Natacha Rousse, Agnes Mugnier, Jerome Soquet, Valentin Loobuyck, Mohamed Koussa, Thomas Modine, Bruno Jegou, Antoine Bical, Ilir Hysi, Olivier Fabre, François Pontana, Regis Matran, Claire Mounier‐Vehier, David Montaigne

**Affiliations:** ^1^ CHU Lille, Institut Coeur‐Poumon Lille France; ^2^ University of Lille, CHU Lille, EA 2694 ‐ Santé publique: épidémiologie et qualité des soins Lille France; ^3^ University of Lille, CHU Lille, Inserm U1008 Lille France; ^4^ University of Lille, CHU Lille, Inserm U1011 Lille France; ^5^ Department of Cardiac Surgery of Artois Centre Hospitalier de Lens et Hôpital Privé de Bois Bernard, Ramsay Générale de Santé Lens France; ^6^ CHU Lille, Department of Clinical Physiology & echocardiography Univ. Lille, Inserm U1011‐EGID Lille France

**Keywords:** aortic dissection, cardiopulmonary exercise testing, prognosis

## Abstract

**Background:**

Although recommendations encourage daily moderate activities in post aortic dissection, very little data exists regarding cardiopulmonary exercise testing (CPET) to personalize those patient's physical rehabilitation and assess their cardiovascular prognosis.

**Design:**

We aimed at testing the prognostic insight of CPET regarding aortic and cardiovascular events by exploring a prospective cohort of patients followed‐up after acute aortic dissection.

**Methods:**

Patients referred to our department after an acute (type A or B) aortic dissection were prospectively included in a cohort between September 2012 and October 2017. CPET was performed once optimal blood pressure control was obtained. Clinical follow‐up was done after CPET for new aortic event and major cardio‐vascular events (MCE) not directly related to the aorta.

**Results:**

Among the 165 patients who underwent CPET, no adverse event was observed during exercise testing. Peak oxygen pulse was 1.46(1.22‐1.84) mlO2/beat, that is, 97 (83–113) % of its predicted value, suggesting cardiac exercise limitation in a population under beta blockers (92% of the population). During a follow‐up of 39(20‐51) months from CPET, 42 aortic event recurrences and 22 MCE not related to aorta occurred. Low peak oxygen pulse (<85% of predicted value) was independently predictive of aortic event recurrence, while low peak oxygen uptake (<70% of predicted value) was an independent predictor of MCE occurrence.

**Conclusion:**

CPET is safe in postaortic dissection patients should be used to not only to personalize exercise rehabilitation, but also to identify those patients with the highest risk for new aortic events and MCE not directly related to aorta.

## 1 INTRODUCTION

Acute aortic dissection is a rare and life‐threatening condition, with poor mid‐term prognosis despite improvement in acute treatment. Post discharge management is based on optimal blood pressure control and aortic imaging follow‐up.^1^ Regular physical activity is a key point of blood pressure management and cardiovascular fitness in both primary and secondary care of cardiovascular diseases.^2^, ^3^ Although recommendations encourage daily moderate activities in post aortic dissection, the safety and benefit of physical activity in this specific context has been poorly explored.^4^, ^5^ While moderate physical activity could be beneficial by lowering blood pressure and thus preventing aortic disease progression, intensive exercise is feared to aggravate aortic expansion. Importantly since promoting physical activity in post aortic dissection patients might be difficult because of their very low aerobic capacities,^6^ cardiopulmonary exercise testing (CPET) represents an affordable tool to evaluate individual fitness and thus personalize patient advices to their daily life activities.^7^ Moreover, CPET might provide prognostic insights in post aortic dissection as it does in several severe medical conditions (e.g. end‐stage heart failure, chronic respiratory failure, end‐stage liver disease).^8^, ^9^ In line we aimed at testing the prognostic significance of CPET regarding aortic and cardiovascular events by exploring a cohort of patients followed‐up after acute aortic dissection.

## 2 METHODS

### 2.1 Study population

Once optimal blood pressure control was obtained, patients referred to our department to plan clinical and morphological follow‐up after an acute (type A or B) aortic dissection were prospectively included in a cohort between September 2012 and October 2017. Patients who underwent traumatic aortic dissection or penetrating aortic ulcers were excluded. Patients with type A aortic dissection who underwent medical treatment solely were also excluded. Intramural aortic hematomas were considered as aortic dissections with total false lumen thrombosis. Connective tissue disease was diagnosed in case of positive Ghent criteria for Marfan syndrome, annulo‐aortic ectasia or SMAD3 mutation. Data collection was approved by the French national data protection commission (Commission Nationale de l'Informatique et des Libertes; CNIL reference #DEC2015‐19). Each patient received written notice regarding his/her rights to withdraw permission for subsequent use of his/her personal data.

### 2.2 Clinical data and blood pressure recording

Clinical characteristics and medication were collected at inclusion. 24‐h blood pressure monitoring was performed and allowed to ensure proper control if blood pressure: blood pressure measure was performed every 15 min with a Spacelabs Medical 90 207 ambulatory blood pressure monitor (Spacelabs Medical Ltd. Issaquah, Wa). In case of aortic stenting, the cuff was placed on the right arm. Medical treatment was recorded at the time of blood pressure monitoring.

### 2.3 Morphological data

Comprehensive transthoracic echocardiography was performed by senior physicians in all patients. Left ventricular ejection fraction was estimated with Simpson's rules. Left ventricle and left atrium dimension were measured according to American Society of Echocardiography. Left ventricular mass was measured according to the American Society of Echocardiography and normalized against body surface area.^10^ Left ventricular filling pressure was considered elevated if E/A mitral inflow ratio was>2 or E/é ratio > 14.^11^ An angio‐CT scan confirmed an aortic diameter below the surgical indication. CT angiograms were realized with an acquisition from the apex of the lungs to the femoral heads. The maximal diameter of the descending aorta behind the left pulmonary artery was used according the Kato's technic.^12^

### 2.4 Cardiopulmonary exercise testing

CPET was performed under antihypertensive drugs including beta‐blocker. CPET was performed in standard manner on a cycle ergometer and firstly aimed at personalizing patient rehabilitation. Patients were encouraged to exercise to exhaustion (symptom‐limited maximal test). Blood pressure was measured every 2 min. A 12‐lead electrocardiogram was monitored during exercise test. Incremental exercise tests were performed on a calibrated electromagnetically braked cycle ergometer (ER‐900; Jaeger, Hochberg, Germany), using a 1‐min step protocol at 10 W min − 1 until exhaustion. Maximal incremental exercise was performed with the subjects maintaining a pedaling frequency of 60 ± 5 revolutions per min. Peak exercise was defined as the highest work level reached during incremental exercise test. During testing, continuous noninvasive monitoring of heart rate and blood pressure was done (Marquette electrocardiogram system, Marquette Electronics, WI, USA). CPET was performed with a metabolic cart (Oxycon Pro, Viasys, France). Continuous measurement of inspired and expired oxygen and carbon dioxide output were done with inline sensors. Peak O2 pulse was calculated by dividing peak oxygen uptake (VO2) by heart rate at the time of peak VO2. The quality of effort was assessed by the respiratory exchange ratio, that is, maximal exercise was defined when peak respiratory exchange ratio > 1.05. Hemoglobin level was checked at the time of exercise testing realization.

### 2.5 Clinical follow‐up

Events were recorded after CPET. Clinical follow‐up was obtained by regular phone calls of patients, as well as interview with their cardiologist and general practitioner, and review of medical files. A new aortic event was defined as a new dissection, aortic rupture, or new scheduled surgical or endovascular procedure, and was the primary endpoint of the study. A scheduled aortic intervention was performed in case of rapid aortic expansion (>5 mm per year) or if aortic diameter reached 55 mm during follow‐up. A secondary endpoint was defined as a composite criterion of major cardiovascular events (MCE) not directly related to aortic event, that is, cardiovascular death, stroke, acute coronary syndrome, hospitalization for cardiac failure and coronary or peripheral artery revascularization.

### 2.6 Statistical analysis

Descriptive analysis was first performed. Numerical parameters were expressed as mean ± standard deviation for normal distribution, median with the interquartile range otherwise. The normality of the distribution was verified by the Shapiro–Wilk test. Qualitative parameters were presented in percentages. Then, survival univariate analyses were performed in order to establish the relationship between risk factors and the time of occurrence of the event. Survival estimates were computed using the Kaplan–Meier method. Comparisons of survival curves according to subgroups were performed with the log‐rank test. The relationship between numerical parameters and survival were tested using the Cox model and optimal thresholds, optimizing the hazard ratio, were determined for parameters reaching a *p* < 0.20. Finally, stepwise Cox model was used to select the best subgroup of variables. Proportional hazards hypothesis and residual analysis were also performed to check the validity of the model. Statistical analyzes were performed with SAS V9.4 software. (Cary, NC, USA).

## 3 RESULTS

**FIGURE 1:**
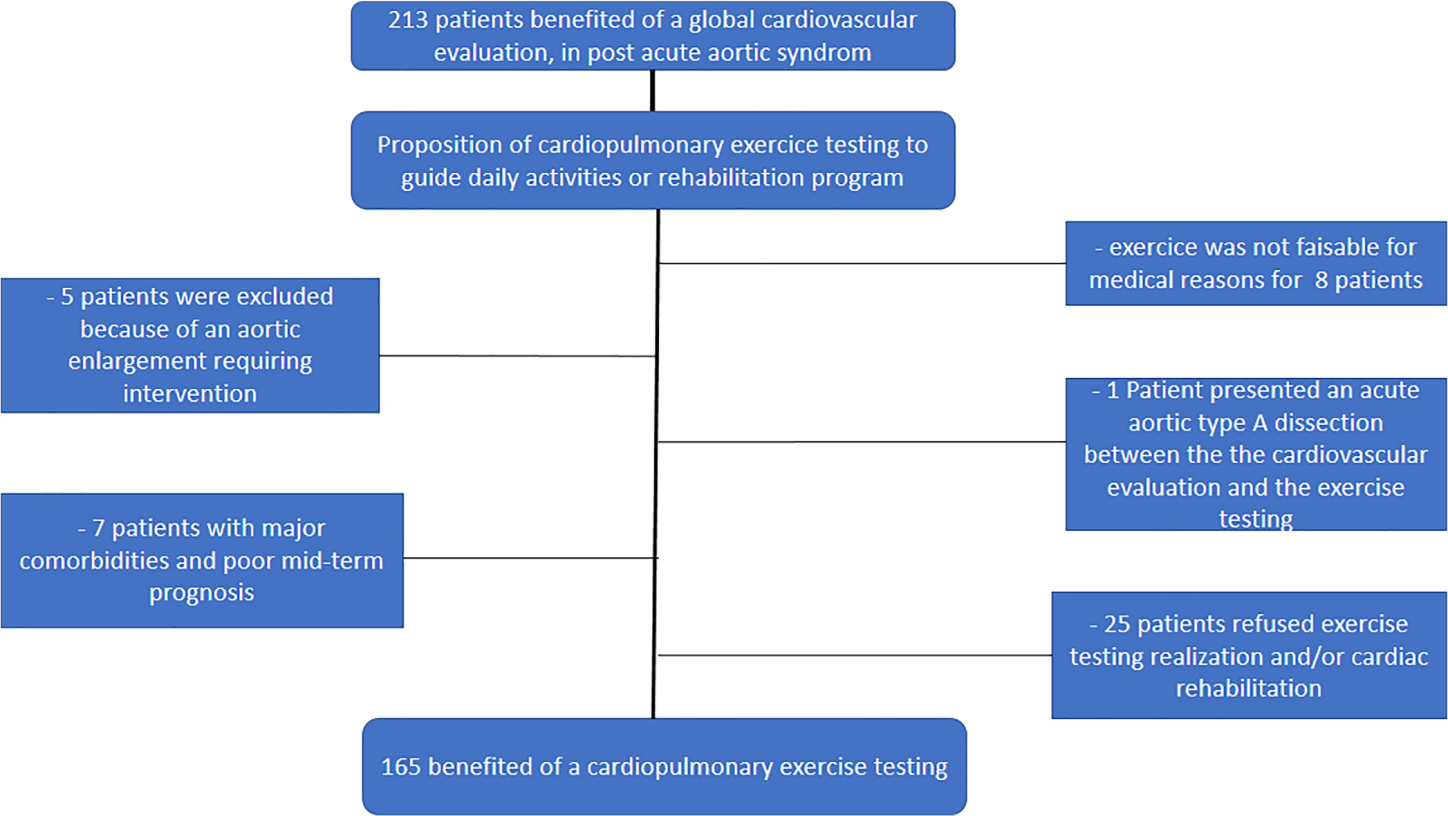
Flow chart of the study population

Among the 213 patients referred to our hospital for post aortic dissection between September 2012 and October 2017, 165 patients underwent CPET. Median delay between the acute aortic event and CPET was 6^4^, ^5^, ^6^, ^7^, ^8^, ^9^, ^10^, ^11^, ^12^, ^13^, ^14^, ^15^, ^16^, ^17^, ^18^, ^19^, ^20^ months. No adverse event was observed during exercise. Flow‐chart of the study is presented in Figure 1 and patient characteristics at inclusion in Table 1. Most patients were middle age men with 16 (9.6%) displaying medical history of connective tissue disease. In line with guidelines, beta‐blockers were taken by the vast majority of patients (92%). Blood pressure was properly controlled under a median of 3 anti‐hypertensive drugs. The ratio of type B: type A aortic dissection was of 1:2. In type B aortic dissection patients, 43 (50%) underwent endovascular therapy (aortic stenting). In type A aortic dissection patients, 40 (50%) patients underwent Bentall aortic surgery, while 40 (50%) patients underwent valve‐sparing ascending aorta replacement. A supplementary aortic arch replacement (partial or total) was also performed in 20 of these type A aortic dissection patients. No left ventricular dysfunction or significant valvular disease was detected on TTE performed before CPET. Echocardiographic data are detailed in Table 2. The median size of the thoracic descending aorta, a strong predictor of aortic events during follow‐up, was 38.3(32–44.6) mm. The thoracic false lumen was totally thrombosed in 45 patients (38.1%).

**TABLE 1:** Baseline characteristic

	All patients	O2 pulse < 85% predicted value	O2 pulse ≥ 85% predicted value
	*n* = 165	*n* = 45	*n* = 117
Clinical			
Age, years	58.2 ± 13.4	55.6 ± 12.4	58.9 ± 12.7
Male, n (%)	116 (70.3)	41 (91)^a^	72(61)
BMI, kg/m2, median (IQR)	26.7 (23.8–30.7)	26.7 (23.0–29.5)	27.0 (24‐31.4)
Abdominal circumference, cm	103.9 ± 4.9	105.9 ± 15.8	103.2 ± 14.8
Current smoking, n (%)	43 (26.0)	14 (31)	29 (25)
Diabetes mellitus, n (%)	10 (6.0)	4 (8)	6 (5)
Dyslipidemia, n (%)	52 (31.7)	13 (28)	39 (33)
Prior atherosclerotic cardiovascular disease, n (%)	26 (15.7)	9 (20)	16 (14)
Type A/B aortic dissection, n	80/85	19/26	58/59
Biological data			
Hemoglobin, g/dl	12.9 ± 1.9	12.9 ± 1.97	12.9 ± 1.85
GFR, ml/mn/1,73 m^2^, median (IQR)	84 (72–103)	87.0 (72–103)	84 (73–103)
LDL cholesterol, g/l, median (IQR)	1.0 (0.76–1.23)	0.94 (0.69–1.09)	1.03 (0.77–1.29)
Blood pressure and treatments 'data			
24‐h SBP, mmHg, median (IQR)	122 (112–133)	124 (116–140)	120 (109–132)
24‐h DBP, mmHg, median (IQR)	70.0 (65–77)	71 (67–78.5)	70 (65–75)
Treatment score, number of hypertensive drugs, median (IQR)	3.0 (2.0–4.0)	3.0 (2.0–4.0)	3.0 (2.0–4.0)
Beta blocker, n (%)	152 (92)	42 (93)	110 (94)
RAAS inhibitor, n (%)	128 (77)	33 (73)	95 (81)
Calcium channel blocker, n (%)	104 (63)	27 (60)	77 (65)
Thiazide diuretic, n (%)	47 (28)	9 (20)	38 (32)
Aldosterone antagonist, n (%)	26 (15)	6 (13)	20 (17)
Statin, n (%)	112 (68)	27 (60)	85 (72)
Morphological data			
Descending aorta diameter at discharge, mm	38.3 (32–44.6)	42 (37.4–48.6)	37.4 (31. 9‐43.5)

Abbreviations: BMI, body mass index; DBP, diastolic blood pressure; GFR, glomerular filtration rate, LDL, low density lipoprotein, RAAS, renin‐angiotensin‐aldosterone system, SBP, systolic blood pressure. Values are expressed as mean (standard deviation) unless otherwise as indicated.

*Note*: Heart rate at peak was missing for three patients, peak exercise oxygen pulse data were thus available for 162 patients.

^a^ *p* < 0.05 vs. patients with O2 pulse≥85% predicted value.

**TABLE 2:** Echocardiography and cardiopulmonary exercise testing (CPET)

	Total population	O2 pulse<85% predicted value	O2 pulse ≥85% predicted value
	*n* = 165	*n* = 45	*n* = 117
Echocardiographic data			
LV ejection fraction, %	63 [60–66]	65 [5 5‐69]	62 [60–66]
LV end‐diastolic diameter, mm	48 [45–62]	50 [46–54]	48 [45–51]
LV end‐diastolic volume, m/m^2^	57 [48–66]	60.2 [48.5–70.0]	57 [48–65]
Stroke volume, ml	69.4 [55. 4‐82.6]	70.1 [57. 4‐84.3]	68.7 [55. 1‐82.5]
LV mass, g/m^2^	94 [76–110]	104 [81–117]	89 [76–108]
Left atrial volume, ml/m^2^	35 [27–43]	35 [26‐42]	35.5 [29–43]
Elevated LV filling pressure, n (%)	13 (8)	2 (4)	11 (9)
Before exercise			
Systolic blood pressure at rest, mmHg	125 [11 5‐136.5]	126 [116–144]	125 [11 5‐135]
Diastolic blood pressure at rest, mmHg	75 [68–83]	76 [65–85]	74.5 [68.5–83.0]
Heart rate at rest, bpm	66 [5 9‐78]	75 [65–92]	65 [58–73]
Exercise data			
Maximum Workload, W	105 [85–140]	110 [75–140]	105 [90–140]
METS	5.5 [4.5–6.7]	5.4 [3.9–6.5]	5.5 [4. 7‐6.7]
Percentage of predicted maximal Workload, %	77 [6 5‐98]	69 [52.5–84]^a^	83 [72–102]
Respiratory exchange ratio at peak	1.20 ± 0.13	1.2 ± 0.17	1.19 ± 0.12
Percentage of predicted peak oxygen uptake, %	75 [63–87]	62 [51–72]^a^	80 [70–91]
Peak oxygen uptake, ml/Kg/mn	18.8 ± 5.21	17.7 ± 5.3	19.1 ± 5.1
Peak oxygen uptake, L/mn	1.46 [1. 22‐1.84]	1.39 [1.09–1.84]	1.5 [1. 23‐1.84]
Percentage of predicted O2 pulse, %	97 [83–113]	76 [72–79]^a^	106 [95–119]
Oxygen pulse, mlO2/beat	11.69 [9.7 9‐14.82]	10.6 [8.9–12.5]^a^	12.5 [10. 1‐15.2]
VE/VCO2 peak	36 [33–40]	36 [3 4‐43]	36 [3 2‐39]
Systolic blood pressure at Peak, mmHg	183 [16 2‐201]	195 [167–205]	180 [160–200]
Diastolic Blood pressure at peak, mmHg	85 [73–96]	86.5 [72–97]	85 [74–95]
Heart rate at peak, bpm	123.9 [120.4–127.3]	134.7 [127.2–142.3]^a^	119 [116.2–123.6]
Percentage of predicted maximum heart rate (Astrand formula), %	76.5 ± 12.7	82.1 ± 14.7^a^	74.4 ± 11.1
Percentage of predicted maximum heart rate (Brawner formula), %	91 [76–106]	99.1 ± 27.8^a^	89.9 ± 20.4
Breathing Reserve	37.91 ± 17.17	39.7 ± 15.5	37.1 ± 17.8

Abbreviations: Bpm, beats per min, LV, left ventricular, METS, metabolic equivalents, VE, ventilation; VCO, carbon dioxide output.

*Note*: Heart rate at peak was missing for three patients; peak exercise oxygen pulse data were thus available for 162 patients.

^a^ *p* < 0.05 vs. patients with O2 pulse≥85% predicted value.

Per‐exercise hemoglobin level was normal in the entire population (12.9 ± 1.8 g/dl). Resting heart rate and blood pressure were within the expected ranges in a population under beta‐blockers and optimized anti‐hypertensive drugs. Details of exercise testing data are provided in Table 2. Peak respiratory exchange ratio was 1.2 ± 0.13 reflecting maximal exercise. Aerobic capacities were globally altered with a mean peak oxygen uptake of 18.8 ± 5.1 ml/Kg/min, corresponding to 75(63–87) % of predicted values and 5.5(4. 5‐6.7) METS. Heart rate at peak exercise was missing for three patients, so peak exercise oxygen pulse data were available for 162 patients. Peak oxygen pulse was 1.46(1. 22‐1.84) mlO2/beat, that is, 97 (83–113) % of its predicted value, suggesting cardiac exercise limitation in a population under beta blockers. No ventilation limitation was observed with a ventilatory reserve at peak exercise >20% in the whole population.

Median follow‐up from CPET was 39(20‐51) months. During follow‐up, 42 aortic event recurrences occurred within 3.7(1. 8‐5.35) months from CPET: 1 aortic rupture, 4 new onset of aortic dissection, 37 new aortic procedures, that is, four ascending aorta interventions, 20 thoracic (arch or isthmus aortic) interventions, one abdominal aortic surgery and 12 aortic stenting of the thoracic descending aorta. Associations between patient characteristics at inclusion and new aortic event risk are presented in Supplementary Table 1. Optimal threshold of event predictors was determined for numerical parameters reaching a *p* value <0.20. A threshold value to best predict event free‐probabilities was defined using the algorithm of maximization of Hazard ratio. The optimal cut‐off to predict aortic event recurrence were respectively 85% for the predictive oxygen pulse value, 50 mm for the left ventricular end‐diastolic diameter, 70% for the predictive peak oxygen uptake and 40 mm for the descending aorta diameter.

**FIGURE 2:**
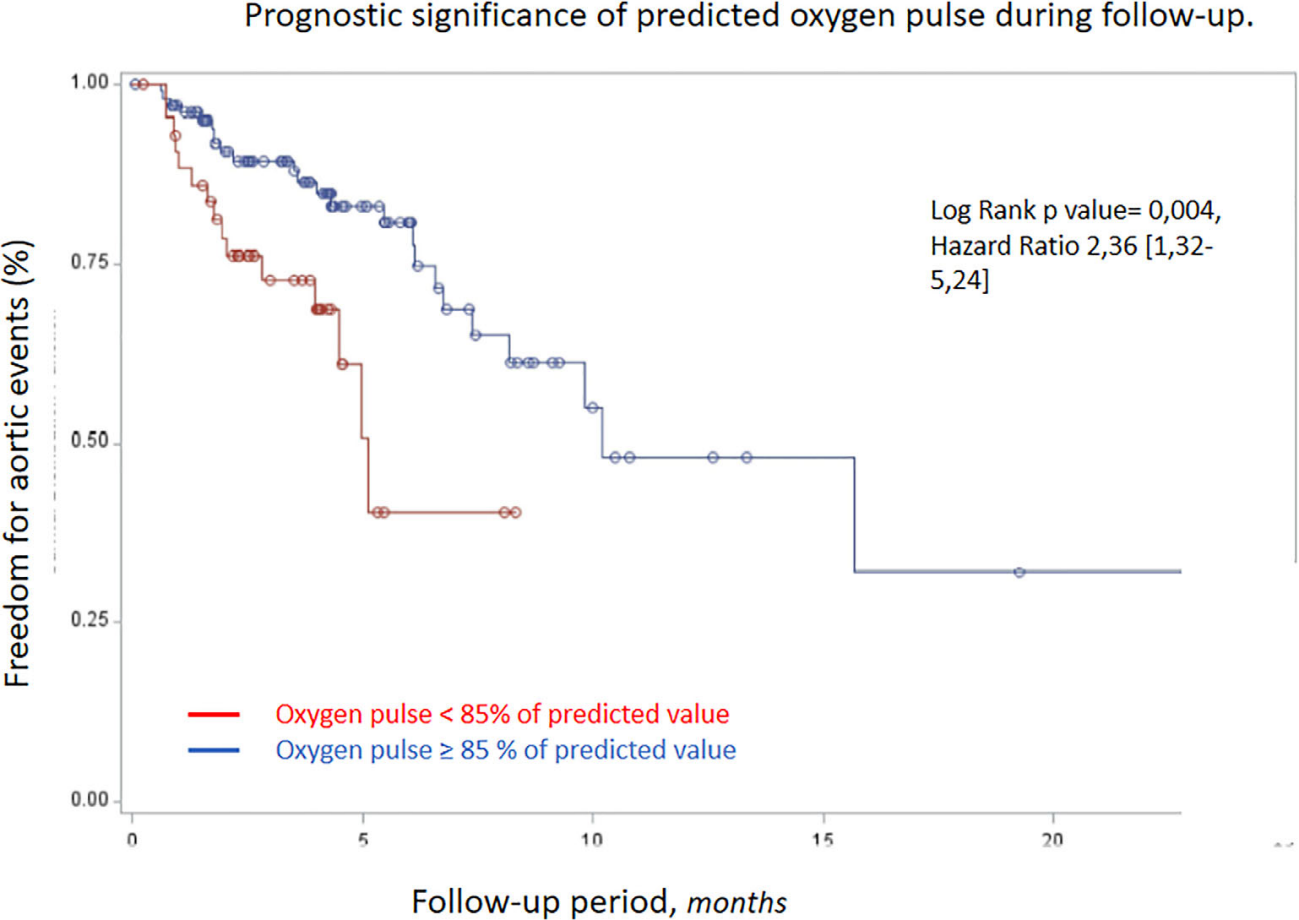
Prognostic significance of oxygen pulse regarding new aortic events

Peak oxygen pulse <85% of predicted value, left ventricular end‐diastolic diameter > 50 mm and thoracic descending aorta diameter > 40 mm were identified by multivariable analysis as independent predictors of aortic event recurrence during follow‐up (Table 3). The association between the risk of aortic events during follow‐up and of peak oxygen pulse is illustrated in Figure 2.

**TABLE 3:** Independent predictors of events. Univariate and multivariate Cox analysis

Variables	Univariates analysis			Multivariate Analysis		
Risk of new aortic events during follow‐up						
	Hazard Ratio	Chi‐Square	p	Hazard Ratio	95% CI	p
Age	0.99	0.41	0.51			
Gender		4.91	0.026			
Stroke Volume	1.01	2.12	0.14			
Hemoglobin	1.06	0.52	0.47			
Percentage of predictive maximal Workload	0.99	1.87	0.17			
LVED diameter > 50 mm		5.73	0.016	**2.81**	**[1.41–5.60]**	**0.003**
Descending aorta diameter > 40 mm		13.37	0.0003	**3.42**	**[1.58–7.36]**	**0.001**
Peak oxygen uptake<70% predicted value		7.59	0.0059			
Peak oxygen pulse<85% predicted value		8.13	0.004	**2.35**	**[1.09–5.04]**	**0.027**
Type A aortic dissection		0.37	0.54			
Risk of nonaortic major cardiovascular events during follow‐up						
	Hazard Ratio	Chi‐Square	p	Hazard ratio	95% CI	p
Age	0.99	0.08	0.76			
Gender		3.42	0.06			
Smoking		0.91	0.63			
24‐h SBP > 135 mmHg		9.94	0.0016	**4.61**	**[1.76–12.1]**	**0.002**
Percentage of peak oxygen pulse	0.98	2.08	0.15			
Peak oxygen uptake<70% predicted value		7.59	0.0059	**4.31**	**[1.63–11.3]**	**0.003**
Type A aortic dissection		3.79	0.05			

Abbreviations: LVED, left ventricular end‐diastolic diameter; SBP, systolic blood pressure.

**FIGURE 3:**
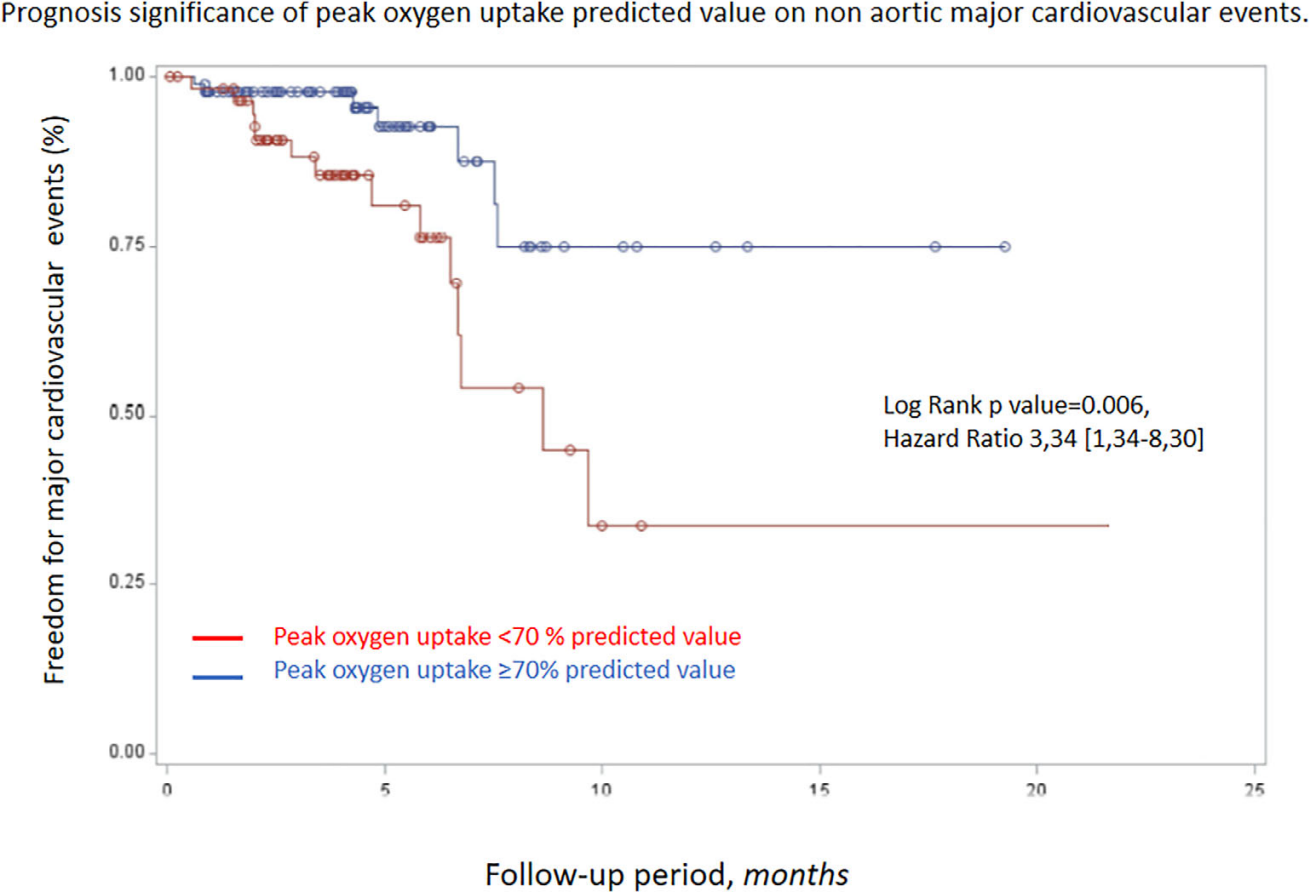
Prognosis significance of peak oxygen uptake regarding nonaortic major cardiovascular events

MCE not related to aortic events occurred in 22 patients during follow‐up: three cardiovascular deaths, eight strokes, one acute coronary syndrome, three hospitalizations for cardiac failure and seven scheduled arterial revascularizations. Predicted peak oxygen uptake and 24‐h systolic blood pressure were associated with MCE onset, with thresholds of 135 mmHg for the 24‐h systolic blood pressure and 70% of predicted peak oxygen uptake identified as the best cut‐off values to predict events using the algorithm of maximization of the Hazard ratio. Interestingly, peak oxygen uptake <70% of predicted value and 24‐h systolic blood pressure > 135 mmHg were the sole independent prognostic factor associated with MCE occurrence (Table 3). The association between the risk of MCE during follow‐up and peak oxygen uptake is illustrated in Figure 3.

## 4 DISCUSSION

Exploring clinical significance of CPET in postaortic dissection syndrome, we confirmed that CPET is safe in patients with well‐controlled blood pressure and can thus be used to tailor and personalize exercise rehabilitation. We also showed that peak exercise oxygen pulse and oxygen uptake are independent predictors respectively of the risk of new aortic events and MCE not directly related to aortic events.

Management of patients surviving aortic dissection is mainly focused on blood pressure control thanks to intensive anti‐hypertensive drugs. Guidelines encourage daily moderate activities for these patients to improve global cardiovascular health and blood pressure control^1^, ^5^: exercise at an intensity of 3 to 5 METs for at least 30 min on most days of the week, for a total of 150 min/week or more. In our study, most of patients had very limited aerobic capacity with maximum workload very close or even lower than 5 METS. The recommended daily practice of moderate exercise at 3–5 METS should thus be adapted and personalized to each patient thanks to CPET.^6^ Aggravation of aortic expansion is feared after intense exercise. We performed CPET in >150 patients under beta‐blockers without any complication, confirming the safety of CPET in those patients with proper control of blood pressure.^13^, ^14^

Mechanisms influencing aortic dilatation and cardiac events in postaortic dissection patients are poorly explored. Aortic evolution is understood as being dependent of aorta properties and systemic factors such as blood pressure control.^15^ In line, we showed that the descending aorta size was associated with a higher risk of aortic event during follow‐up. We also highlighted that oxygen pulse is related to aortic events. Oxygen pulse is the product of stroke volume and arteriovenous oxygen difference during exercise, and thus an indicator of cardiac performance in the absence of anemia.^16^ Thus, oxygen pulse increases linearly with increasing workload until it reaches its highest value, that is, a kinetic similar to stroke volume raise.^17^ We interpret abnormal peak oxygen pulse in our study as an index of impaired cardiovascular performance secondary to elevated aortic‐related afterload.^18^ Indeed, abnormal pulsatile aortic loading during exercise can occur in postdissection aorta independent of hypertension because of thrombosed false lumens, abnormal aortic compliance and pulse wave reflection. Ventriculo‐arterial uncoupling despite well‐controlled blood pressure would develop with stress and reflect arterial tree burden. This hypothesis should be tested further thanks to noninvasive evaluation of ventriculo‐arterial coupling during exercise.^19^, ^20^

Oxygen pulse is also linked to arteriovenous difference during exercise. This parameter was not directly evaluated in our observational study since its assessment requires invasive measurements. Although peripheral oxygen extraction at peak exercise is influenced by the level of physical activity, this phenomenon is of limited impact in nonathlete individuals.^21^ Finally we can also hypothesized that a lower oxygen pulse reflected higher sympathetic activity. A higher sympathetic activity increases heart rate at rest and during exercise. Different situations associated with high sympathetic activity are known to influence aortic root size, aortic aneurysm expansion and also influence aortic expansion following in post aortic dissection. Obesity is associated with aortic size at different level and influence aortic enlargement. Sleep apnea syndrome, which was not specifically explored in our study, has been shown to be very prevalent in the population of patients suffering from aortic dissection and is associated with an oversympathetic activity.

Our findings have potential clinical implications. If clinical benefits of cardiac rehabilitation post aortic dissection has not been evaluated well so far, cardiac rehabilitation activities seems to be beneficial in this context. In a small sample size study, peak oxygen consumption was significantly increased after a cardiac rehabilitation program.^22^ Enhancement of peak oxygen uptake has already shown its benefit on cardiovascular secondary prevention in atherosclerotic populations. Our work comfort the hypothesis that cardiac rehabilitation activities following by moderate chronic exercise should be of interest in post aortic dissection. Mas‐Stachurska and al showed in a murine model of Marfan syndrome that moderate physical activity tends to decelerate aortic dilatation rate and improve cardiac hemodynamic overload. These results reflect the benefit of moderate physical activity. Physical activities recommendations must be advised with caution, intensive physical activity have probably deleterious effect.^23^ By increasing peak oxygen uptake and decreasing maximal heart rate at exercise, regular physical activities are expected to improve peak oxygen pulse. We can thus hypothesize that regular activity could reduce residual aortic and cardiovascular risk. Moreover, regular physical activity is an integrant part of lowering blood pressure and weight loss.

Aerobic capacity was evaluated for the first time in our study in postaortic dissection to stratify the risk of cardiovascular events. Peak oxygen uptake predicted nonaortic cardiac events. CPET is thus shown to be of prognostic value, along the same line as in several other chronic medical conditions.^24^ Aerobic capacity evaluation might be implemented in post aortic dissection management to only to guide cardiac rehabilitation but also to stratify cardiovascular risk.^25^, ^26^

This work must be read with its limits, as the single center observational design. The exercise testing was performed once during follow‐up, and data on patient's physical activities before and after acute aortic dissection were not collected. The population is quite heterogeneous with different specific management in the aortic dissection acute phase. Exploration of sleep apnea syndrome and sympathetic hyperactivity would have brought interesting data regarding the interplay between neurovegetative system, blood pressure and outcomes. These explorations were not performed and would need further research.”

In conclusion, the risk of recurrent aortic event remains high after aortic dissection. On top of personalization of exercise rehabilitation, CPET helps stratifying the risks of new aortic events and MCE not directly related to aortic events in postaortic dissection patients. The influence of aerobic capacity enhancement on residual risk following an aortic dissection needs to be further investigated. Cardiac rehabilitation program appears to be essential following aortic dissection.

## CONFLICT OF INTEREST

The authors declare no conflicts of interest regarding this work.

## Supporting information


**Supplementary Table 1** Predictors of aortic events during follow‐up. Univariate Cox analysis.

## Data Availability

The data that support the findings of this study are available from the corresponding author upon reasonable request.
