# Supplementary material for: Prognostic value of aerobic capacity and exercise oxygen pulse in postaortic dissection patients
Source: Clin Cardiol. 2020 Dec 31;44(2):252–60. doi: 10.1002/clc.23537 (PMC7852169; doi:10.1002/clc.23537)
Supplement: Supplementary file 1 — Supplementary Table 1 Predictors of aortic events during follow‐up. Univariate Cox analysis. [file CLC-44-252-s001.docx]

Supplementary Table 1. Predictors of aortic events during follow-up. Univariate Cox analysis.

| Qualitative parameters | | | | |
| --- | --- | --- | --- | --- |
| Variables | Beta coefficient | Standard Error | | p |
| General characteristics |  |  | |  |
| Male gender | **0.95** | **0.44** | | **0.032** |
| Current smoking | 0.69 | 0.39 | | 0.08 |
| Diabetes Mellitus | -1.10 | 1.01 | | 0.27 |
| Dyslipidemia | -0.22 | 0.34 | | 0.51 |
| Prior atherosclerotic disease | 0.00 | 0.42 | | 0.99 |
| Type A aortic dissection | -0.19 | 0.32 | | 0.54 |
|  |  |  | |  |
| Morphological data |  |  | |  |
| Thoracic descending aorta diameter> 40 mm | **1.19** | **0.35** | | **0.0006** |
|  |  |  | |  |
| Exercise data |  |  | |  |
| Peak oxygen uptake <70% predicted value | **-0.81** | **0.32** | | **0.012** |
| Peak oxygen pulse<85% predicted value | **0.97** | **0.35** | | **0.006** |
|  |  |  | |  |
| Echocardiographic and data |  |  | |  |
| LV end-diastolic diameter>50 mm | **0.75** | **0.32** | | **0.019** |
| Left atrial enlargement | 0.43 | 0.32 | | 0.18 |
| Left ventricular hypertrophy | 0.01 | 0.37 | | 0.98 |
|  |  |  | |  |
| Numerical Parameters | | | | |
| Variables | Beta coefficient | Standard Error | p | |
| General characteristics |  |  |  | |
| Age, *years* | -0.008 | 0.01 | 0.52 | |
| Body mass index, *Kg/m²* | -0.01 | 0.03 | 0.65 | |
| 24-hour mean SBP, *mmHg* | 0.007 | 0.02 | 1.00 | |
| 24-hour mean DBP, *mmHg* | 0.01 | 0.02 | 0.32 | |
| Treatment score, *n* | 0.03 | 0.12 | 0.79 | |
| Hemoglobin, *g/L* | 0.065 | 0.09 | 0.47 | |
| MDRD glomerular filtration rate, *mL/mn/1.73m²* | 0.007 | 0.006 | 0.24 | |
|  |  |  |  | |
| Morphological data |  |  |  | |
| Thoracic descending aorta diameter, *mm* | **0.06** | **0.01** | **<0.0001** | |
|  |  |  |  | |
| Exercise data |  |  |  | |
| Peak oxygen uptake, *mL/Kg/mn* | -0.02 | 0.03 | 0.56 | |
| Percentage of predicted peak oxygen uptake, *%* | **-0.02** | **0.009** | **0.026** | |
| Peak oxygen pulse, *mL/beats* | 0.005 | 0.04 | 0.89 | |
| Percentage of predicted peak oxygen pulse, *%* | **-0.011** | **0.0077** | **0.15** | |
| Maximum workload, *Watts* | 0.004 | 0.004 | 0.32 | |
| Percentage of predicted workload, *%* | -0.01 | 0.007 | 0.17 | |
| METS | 0.12 | 0.09 | 0.89 | |
| Respiratory exchange ratio | -2.46 | 1.49 | 0.10 | |
| VE/VCO2 slope | -0.06 | 0.02 | 0.80 | |
| Percentage of age-predicted maximal heart rate, *%* | -0.004 | 0.01 | 0.74 | |
|  |  |  |  | |
| Echocardiographic data |  |  |  | |
| LV end-diastolic diameter, *mm* | **0.084** | **0.03** | **0.007** | |
| Stroke volume, *mL* | 0.10 | 0.07 | 0.14 | |
| Left ventricular ejection fraction, *%* | -0.01 | 0.02 | 0.52 | |
| Left ventricular mass, *g/m²* | 0.0007 | -0.005 | 0.87 | |
| Left atrial volume, *mL/m²* | 0.016 | 0.01 | 0.28 | |

**Legend.** DBP: diastolic blood pressure, METS: metabolic equivalents, MDRD: modification of diet in renal disease, SBP: systolic blood pressure.
